# Reduced phagocytosis, ROS production and enhanced apoptosis of leukocytes upon alcohol drinking in healthy volunteers

**DOI:** 10.1007/s00068-021-01643-x

**Published:** 2021-03-30

**Authors:** Florian Haag, Andrea Janicova, Baolin Xu, Maciej Powerski, Melanie Fachet, Katrin Bundkirchen, Claudia Neunaber, Ingo Marzi, Borna Relja, Ramona Sturm

**Affiliations:** 1 Experimental Radiology, Department of Radiology and Nuclear Medicine, Otto Von Guericke University, Magdeburg, Germany; 2 Department of Trauma, Hand and Reconstructive Surgery, Goethe University, Frankfurt, Germany; 3 Chair of Medical Systems Technology, Institute for Medical Technology, Faculty of Electrical Engineering and Information Technology, Otto Von Guericke University, Magdeburg, Germany; 4 Trauma Department, Hannover Medical School, Hannover, Germany

**Keywords:** Alcohol, Granulocytes, Monocytes, Phagocytosis, ROS, Apoptosis

## Abstract

**Background:**

Alcohol drinking is associated with a serious risk of developing health problems as well as with a large number of traumatic injuries. Although chronic alcohol misuse is known to contribute to severe inflammatory complications, the effects of an acute alcohol misuse are still unclear. Here, the impact of acute alcohol drinking on leukocyte counts and their cellular functions were studied.

**Methods:**

Twenty-two healthy volunteers (12 female, 10 male) received a predefined amount of a whiskey-cola mixed drink (40% v/v), at intervals of 20 min, over 4 h to achieve a blood alcohol concentration of 1‰. Blood samples were taken before drinking *T*_0_, 2 h (*T*_2_), 4 h (*T*_4_), 6 h (*T*_6_), 24 h (*T*_24_) and 48 h (*T*_48_) after starting drinking alcohol. Leukocytes, monocytes and granulocyte counts and their functions regarding the production of reactive oxidative species (ROS), phagocytosis and apoptosis were analyzed by flow cytometry.

**Results:**

Total leukocyte counts significantly increased at *T*_2_ and *T*_4_, while granulocyte and monocyte counts decreased at *T*_4_ and *T*_6_ vs. *T*_0_. Monocytes increased significantly at *T*_24_ and *T*_48_ vs. *T*_0_. While the total number of ROS-producing leukocytes and notably granulocytes significantly increased, in parallel, the intracellular ROS intensity decreased at *T*_2_ and *T*_6_. The numbers of ROS-positive monocytes have shown a delayed modulation of ROS, with a significant reduction in the total number of ROS-producing cells at *T*_48_ and a significantly reduced intracellular ROS-intensity at *T*_24_. Phagocyting capacity of leukocytes significantly decreased at *T*_4_ and *T*_6_. In general leukocytes, and notably granulocytes demonstrated significantly increased early (*T*_2_), while monocyte exerted significantly increased late apoptosis (*T*_24_ and *T*_48_).

**Conclusions:**

Alcohol drinking immediately impacts leukocyte functions, while the impact on monocytes occurs at even later time points. Thus, even in young healthy subjects, alcohol drinking induces immunological changes that are associated with diminished functions of innate immune cells that persist for days.

## Introduction

The consumption of alcohol is globally prevalent. In 2018, the World Health Organization’s (WHO) “Global status report on alcohol and health” reported that 43% of the world’s population over the age of 15 consumes alcohol [1]. In particular, the 2018 ESA study showed that 71.6% of Germans surveyed had consumed alcohol in the 30 days prior to the survey [2]. Alcohol consumption promotes the pathogenesis of numerous diseases and increases the risk of being involved in an accident and suffering a trauma [1]. In this context, blood alcohol concentration (BAC) correlates positively with injury severity [3]. The frequently observed changes in the course of diseases in alcohol-consuming individuals may be caused by the effects of alcohol on the immune system. Alcohol impacts both the function and the number of monocytes and neutrophils, which represent an important defense mechanism of the innate immune system against infections [4–6]. Decreased response of monocytes and neutrophils to inflammatory stimuli such as damage-associated molecular patterns (DAMPs) and/or pathogen-associated molecular patterns (PAMPs) is associated with an increased incidence of complications such as sepsis and organ failure, as well as an increased mortality upon infection [7–9]. The effects of alcohol on the immune system show a biphasic characteristic and depends on the time of exposure as well as the dose [10–12]. Within 20 min after alcohol consumption, the number of circulating leukocytes increases, suggesting an early pro-inflammatory response, which is followed by an anti-inflammatory response with a systemic decrease in monocytes and natural killer cells. Also, systemic levels of pro-inflammatory interleukin (IL)-1β, IL-6, and monocyte chemoattractant protein (MCP)-1 decrease while anti-inflammatory IL-10 increases [10, 12, 13]. This later anti-inflammatory effect may cause the development of PAMPs tolerances [14]. This is underlined by reduced tissue invasion, as well as ROS-production and phagocytosis activity of the mentioned cells [5, 15–17]. Additionally, apoptosis mediated by caspases-3 and 7 is of crucial importance for balancing the immune system reactivity between immune suppression and regulation [18, 19]. It has been shown in vivo that neutrophils were less prone to apoptosis during thoracic trauma under the influence of alcohol [16].

These immune-suppressive effects on the immune system may also have an impact on alcoholized trauma patients. In a retrospective analysis of traumatized patients differences in outcomes between patients who were sober at the time of the accident, alcoholized patients, and patients with chronic alcohol abuse (alcoholic liver cirrhosis) were found [20]. In vivo studies also demonstrated that acute alcohol intoxication significantly reduced mortality after hemorrhagic shock [21]. Although in vivo studies indicate that certain anti-inflammatory properties might cause even beneficial effects in conditions of acute inflammation, notably in the human scenario the knowledge is sparse. Thus, to improve the understanding of alcohol`s impact on the immune system, we investigated the functional changes of leukocytes in terms of phagocytosis, ROS-formation and apoptosis upon acute alcohol drinking in healthy volunteers in a time and dose-dependent manner. Furthermore, gender-based differences have been assessed since here even less is known.

## Patients and methods

### Ethics

This study was performed in accordance with the institutional ethics committee approval from the University Hospital of the Goethe-University Frankfurt (No. 255/14), in accordance with the Declaration of Helsinki and following the Strengthening the Reporting of Observational studies in Epidemiology-guidelines [22]. All healthy volunteers (HV) signed the written informed consent form themselves in accordance with the ethical standards after detailed explanation of the investigations.

### Study setting and population

Twelve female and ten male HV between 20 and 37 years of age were included. Exclusion criteria were a history of chronic alcohol consumption and pre-existing chronic inflammatory diseases, immunological disorders, HIV and hepatitis, immune-suppressive or anti-coagulant medication.

### Study protocol

The trial day started each time in the early afternoon with a maximum of three HV per day. After eating a standardized meal, each HV got a 20 G venous cannula (Vasofix Braunüle, Braun, Melsungen Germany). The time when the HV started drinking was determined to be 4 pm. Each HV received over 4 h every 20 min a defined amount of alcohol according to the Widmark equation, including sex, age, high and weight of HV to reach a blood alcohol level of 1‰. The drinks consisted of Tennessee Whiskey Jack Daniels (40%) and cola (Coca-Cola) in a mixing ratio of 1:2. After the drinking period, a 2 h monitoring phase without further alcohol consumption followed. During the experiment, the maximum of additional drinks for the HV was limited by a liter of water. The test person were medically supervised and cared for the entire time. The same setting, population and protocol were used for additional studies which addressed other aspects of the alcohol-caused modifications of the immune system.

### Blood sampling

Blood was then taken from the venous cannula at defined time points, before drinking at *T*_0_, 2 (*T*_2_), 4 (*T*_4_) and 6 h (*T*_6_) after starting the drinking period in heparin tubes (S-Monovette^®^ lithium-heparin, Sarstedt, Nürmbrecht, Germany). Blood was also taken after 24 h (*T*_24_) and 48 h (*T*_48_) on the following days. To determine the BAC blood was taken every hour from *T*_0_ to *T*_48_ in serum-gel tubes (Sarstedt).

### Phagocytosis

To determine the phagocytosis activity of the monocytes and granulocytes, 100 µL heparinized whole blood were incubated with FITC-labelled *E. coli* bacterial solution (*Escherichia coli*, K-12 strain, BioParticles™, Invitrogen, USA) according to the manufacturer's instructions. A negative control without *E. coli* was included. The cells were incubated for 1 h at 37 °C and 5% CO_2_ in darkness. Subsequently, 2 ml FACS buffer were added and samples were centrifuged at 300*g* for 5 min at room temperature followed by incubation in 0.5 ml of BD FACS Lysing Solution at room temperature and protected from light for 10 min. Then, 2 ml of FACS buffer (phosphate-buffered solution, PBS w/o Ca^+^/Mg^+^ plus 0.5% bovine serum albumin, BSA) were added and samples were centrifuged at 300*g* for 7 min at room temperature. This step was repeated one more time and cells were diluted in 100 μl FACS buffer and stored on ice until measurement. The phagocytizing activity of granulocytes and monocytes was quantified as a percentage of absolute cell numbers measured using a commercial flow cytometric analysis by BD FACS Canto II (BD Biosciences, Franklin Lakes, USA). From each sample, a minimum of 50,000 cells was measured, which were subsequently analyzed. Cells were gated according to their forward and side-scatter profiles.

### Reactive oxygen species

The ROS detection reagent CM-H_2_DCFDA from Invitrogen (USA) was used to determine the production of free oxygen species. 100 µl heparinized whole blood were incubated with CM-H_2_DCFDA reagent according to the manufacturer’s instructions. A negative control without reagent was included. Cells were incubated for 30 min at 37 °C and 5% CO_2_ in darkness. After washing with RPMI 1640 medium, another incubation period for one hour at 37 °C and 5% CO_2_ followed. Then, 2 ml FACS buffer were added and samples were centrifuged at 300*g* for 5 min at room temperature followed by incubation in 0.5 ml of BD FACS Lysing Solution at room temperature and protected from light for 10 min. Subsequently, 2 ml of FACS buffer were added and samples were centrifuged at 300*g* for 7 min at room temperature. This step was repeated one more time and cells were diluted in 100 μl FACS buffer and stored on ice until measurement. Since CM-H_2_DCFDA passively diffuses into the cells, the intracellular reactions with subsequent oxidation result in a fluorescent adduct, which enabled flow cytometric analysis using a BS FACS Canto II (BD Biosciences). Leukocytes, monocytes and granulocytes were gated according to their forward- and side-scatter profiles.

### Apoptosis

To investigate apoptosis caspase-3 and 7 were determined. Caspase activity is found in all apoptotic processes and is thus associated with regulated cell death. 50 µl of heparinized whole blood were incubated with the Fluorochrome Inhibitor of Caspases (FLICA) reagent FAM-DEVD-FMK, a target sequence between a green fluorescent label, carboxyfluorescein (FAM) and a fluoromethylketone (FMK) according to the manufacturer’s instructions (FAM-FLICA® Caspase-3/7 Assay Kit, Immuno Chemistry, Bloomington, US). A negative control without FAM-FLICA reagent was included. Cells were incubated for 90 min at 37 °C and 5% CO_2_ in darkness. Then, leukocytes were isolated by lyzing red blood cells and the sample were washed with FACS buffer as described above. The reagent enters the cells and irreversibly binds to activated caspases-3 and 7. The green fluorescent signal is a direct measure of active caspase-3 and 7 enzyme activity and detectable with flow cytometric analysis using a BD FACS Canto II (BD Biosciences). Leukocytes, monocytes and granulocytes were gated according to their forward and side-scatter profiles.

### Statistical analysis

GraphPad prism 6.0 software (GraphPad Software Inc. San Diego, CA, USA) was used to perform the statistical analysis. Data are given as mean ± standard error of the mean (SEM). The Kruskal–Wallis test with a Dunn’s post hoc test was applied to compare the differences between the groups. A *p* value below 0.05 was considered statistically significant.

## Results

### Study population and blood alcohol concentration

Twenty-two healthy volunteers (HV) were enrolled in this study. The mean age was 25 ± 4 years. 54.55% of the patients were female. The BAC increased significantly at *T*_2_ and after 4 h at *T*_4_ the aimed BAC of one per mille was reached. After 24 h as well as after 48 h no BAC was detectable. There were no significant gender-specific differences.

### Alcohol-induced changes in circulating cell numbers of leukocytes

**Fig. 1:**
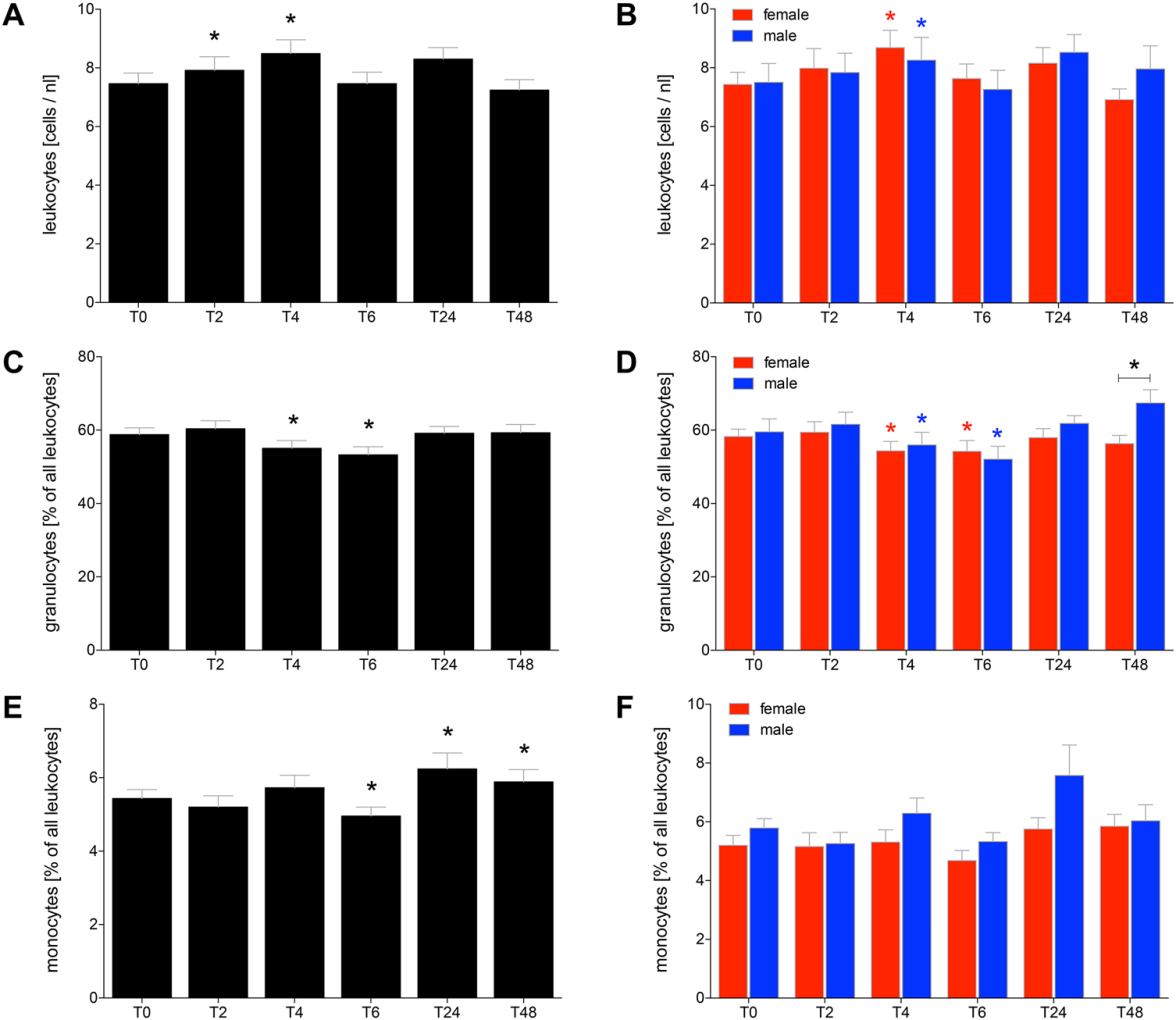
Total leukocyte counts after acute alcohol drinking in healthy volunteers before, during and after drinking (**a**, **b**). The % of granulocytes (**c**, **d**) as well as monocytes (**e**, **f**) out of leukocytes. The analyses were performed in 12 female and 10 male volunteers before *T*_0_, 2 h (*T*_2_), 4 h (*T*_4_), 6 h (*T*_6_), 24 h (*T*_24_) and 48 h (*T*_48_) after the start of alcohol consumption. The data are presented as mean ± standard error of the mean. *p <0.05 vs *T*_0_ or *p *<0.05 vs. indicated groups

The total number of leukocytes significantly increased at *T*_2_ and at *T*_4_ compared with cell counts determined at *T*_0_ before drinking (*p* < 0.05, Fig. 1a). Both female and male had a significant increase in the leukocytes counts at *T*_4_ compared to cell numbers at *T*_0_ before drinking (*p* < 0.05, Fig. 1b).

The proportion of granulocytes out of all leukocytes significantly decreased 4 h after starting of alcohol consumption at *T*_4_ and *T*_6_ compared *T*_0_, respectively (*p* < 0.05, Fig. 1c). This decrease was significant in both female and male HV (*p* < 0.05, Fig. 1d). However, at *T*_48_ there was a significant difference between female and male HV in the proportion of granulocyte, with significantly increased percentage in male (*p* < 0.05, Fig. 1d).

The proportion of monocytes out of all leukocytes significantly decreased at *T*_6_ compared to *T*_0_ (*p* < 0.05, Fig. 1e). At *T*_24_ and *T*_48_ the proportion of monocytes out of all leukocytes significantly increased compared to *T*_0_ (*p* < 0.05, Fig. 1e). There were no significant changes between female and male (Fig. 1f).

### Alcohol-reduced production of reactive oxygen species in leukocytes

**Fig. 2:**
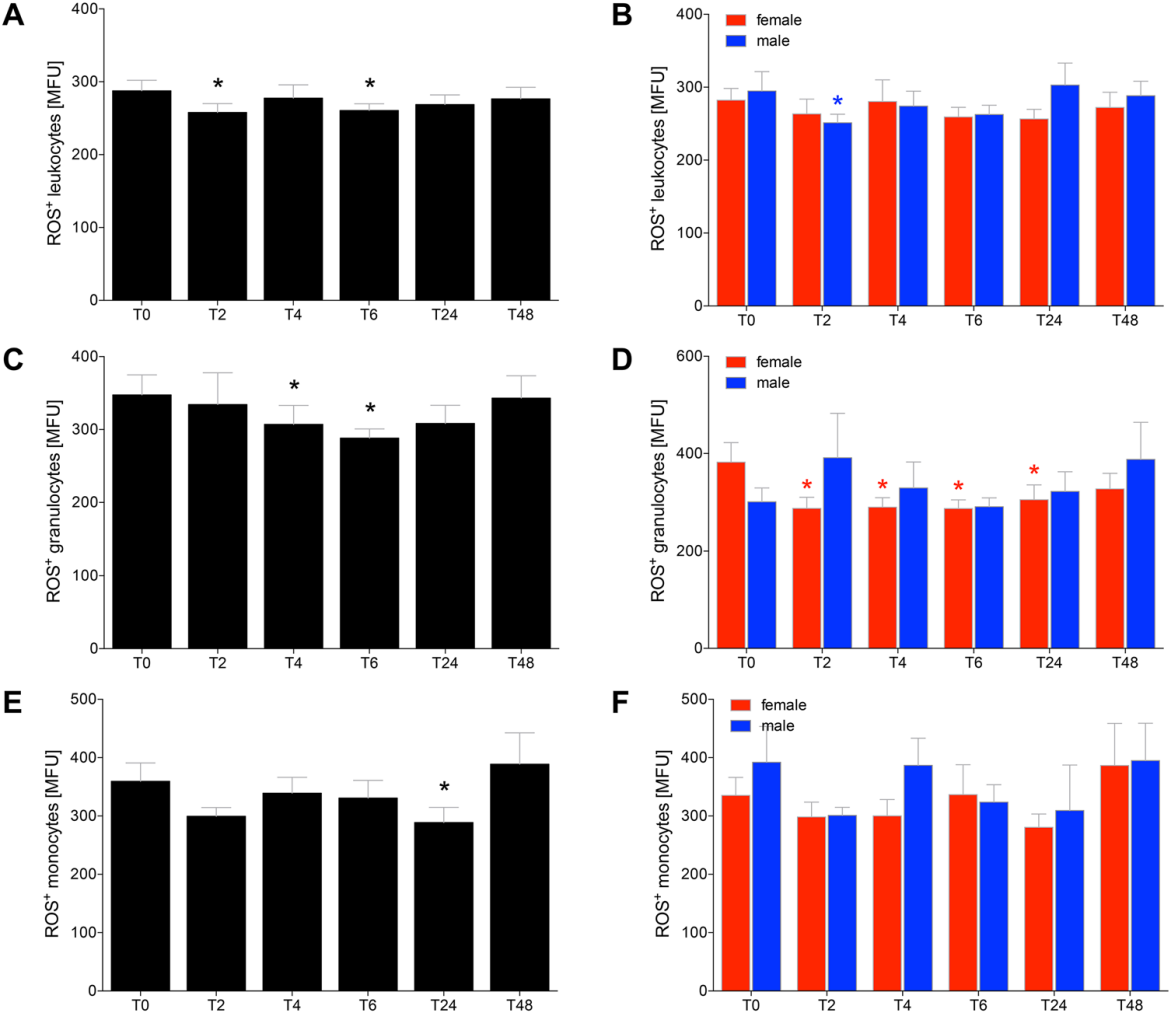
The capacity of cells to produce reactive oxygen species (ROS) after acute alcohol drinking in healthy volunteers before, during and after drinking in leukocytes (**a**, **b**), granulocytes (**c**, **d**) and monocytes (**e**, **f**). The analyses were performed in 12 female and 10 male healthy volunteers before *T*_0_, 2 h (*T*_2_), 4 h (*T*_4_), 6 h (*T*_6_), 24 h (*T*_24_) and 48 h (*T*_48_) after the start of alcohol consumption. The data are presented as the mean of the mean fluorescence intensity (MFU) ± standard error of the mean. *p <0.05 vs. *T*_0_

The ROS-producing capacity of leukocytes significantly decreased at *T*_2_ and *T*_6_ compared to *T*_0_ (*p* < 0.05, Fig. 2a). ROS-producing capacity in leukocytes was significantly decreased in male HV at *T*_2_ compared to *T*_0_ (*p* < 0.05, Fig. 2b).

Considering the ROS-producing capacity of granulocytes, there was a significant decrease at *T*_4_ and *T*_6_ compared to *T*_0_ (*p* < 0.05, Fig. 2c). The comparison between female vs. male HV has shown, that the ROS-producing capacity of granulocytes was significantly decreased at *T*_2_, *T*_4_, *T*_6_ as well as *T*_24_ compared to *T*_0_ in female (*p* < 0.05, Fig. 2d).

The ROS-producing capacity of monocytes was significantly decreased at *T*_24_ compared to *T*_0_ (*p* < 0.05, Fig. 2e). The comparison between female and male HV has shown no significant differences (Fig. 2f).

### Alcohol-reduced phagocytosis in leukocytes

**Fig. 3:**
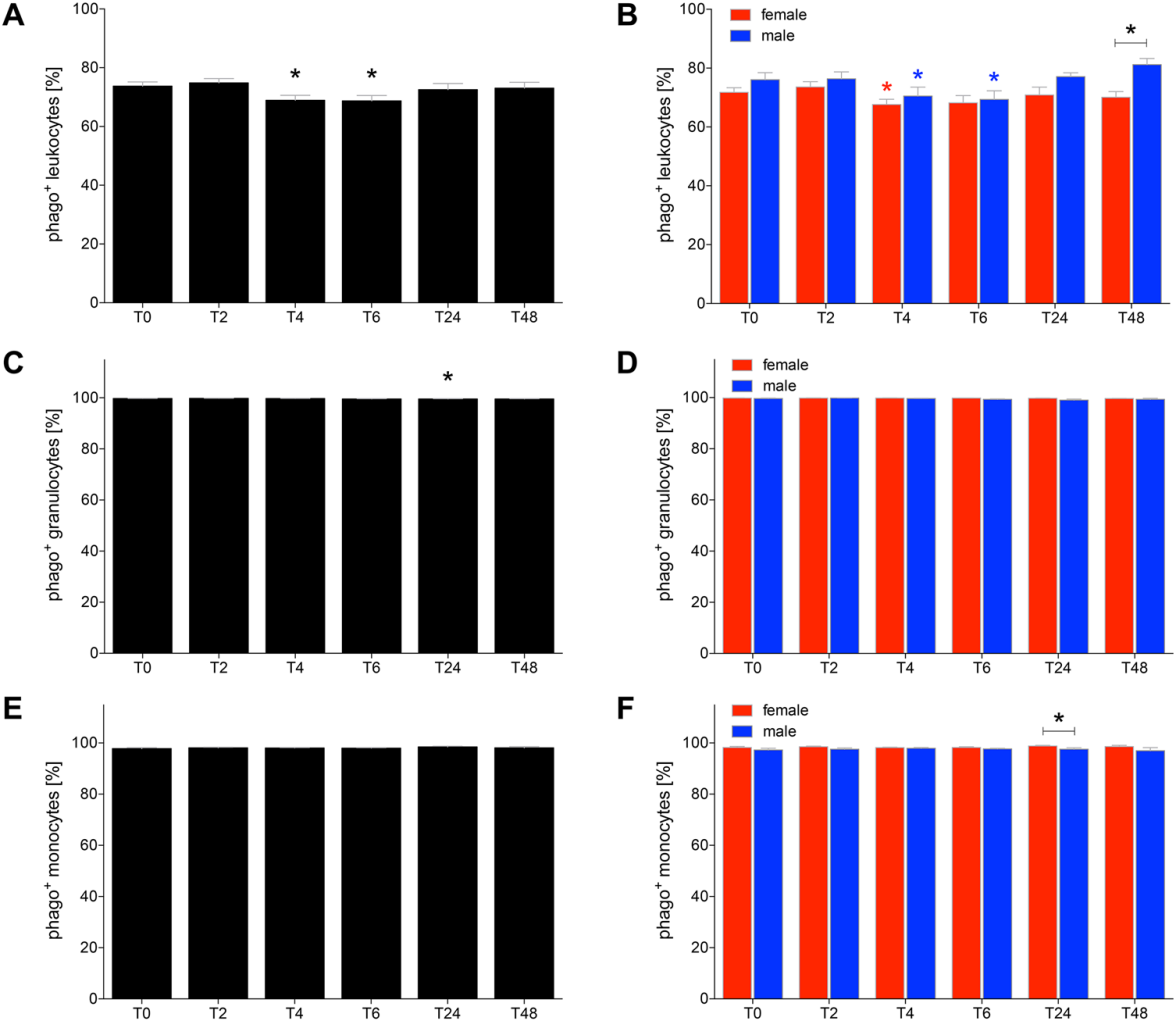
The ratio of phagocytizing leukocytes (**a**, **b**), granulocytes (**c**, **d**) and monocytes (**e**, **f**) after acute alcohol drinking in healthy volunteers before, during and after drinking. The analyses of cells positive for phagocytosis (phago^+^) were performed in 12 female and 10 male healthy volunteers before *T*_0_, 2 h (*T*_2_), 4 h (*T*_4_), 6 h (*T*_6_), 24 h (*T*_24_) and 48 h (*T*_48_) after the start of alcohol consumption. The data are presented as the mean ± standard error of the mean. *p < 0.05 vs. *T*_0_ or *p *< 0.05 vs. indicated groups

The proportion of phagocytizing leukocytes was significantly decreased at *T*_4_ and *T*_6_ compared to *T*_0_ (*p* < 0.05, Fig. 3a). This decrease was significant at *T*_4_ in both female and male HV, while at *T*_6_ the decrease was only significant in male compared to *T*_0_ (*p *< 0.05, Fig. 3b). During the complete observational period female HV had lower proportions of phagocytizing leukocytes compared to men, however, this difference was only significant at *T*_48_ (Fig. 3b).

The proportions of phagocytizing granulocytes remained stable with a slight increase at *T*_24_, while there were no differences among monocytes (Fig. 3c–f).

**Fig. 4:**
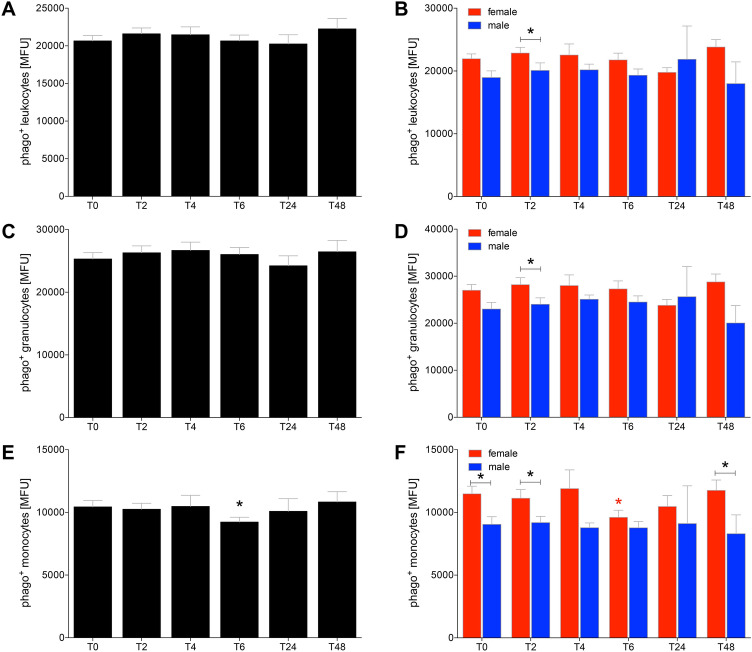
The phagocytizing capacity of leukocytes (**a**, **b**), granulocytes (**c**, **d**) and monocytes (**e**, **f**) after acute alcohol drinking in healthy volunteers before, during and after drinking. The analyses of mean fluorescence units (MFU) as the capacity measure for phagocytosis were performed in 12 female and 10 male healthy volunteers before *T*_0_, 2 h (*T*_2_), 4 h (*T*_4_), 6 h (*T*_6_), 24 h (*T*_24_) and 48 h (*T*_48_) after the start of alcohol consumption. The data are presented as the mean ± standard error of the mean. *p < 0.05 vs. *T*_0_ or *p *< 0.05 vs indicated groups

The phagocytizing capacity was not significantly changed among leukocytes and granulocytes (Fig. 4a–d). In female HV the capacity of phagocytizing leukocytes was in general higher compared to male HV, while this difference was significant only at *T*_2_ (*p* < 0.05, Fig. 4b, d).

The phagocytizing capacity of monocytes was significantly decreased at *T*_6_ compared to *T*_0_ (*p* < 0.05, Fig. 4e). This decrease was only significant in female HV at *T*_6_ compared to *T*_0_ (*p* < 0.05, Fig. 4f). In general, female HV had higher phagocytizing capacity of monocytes compared to male HV at every time point. This gender difference was significant at *T*_0_ before drinking, at *T*_2_ and *T*_48_ (*p* < 0.05, Fig. 4f).

### Alcohol-increased apoptosis in leukocytes

**Fig. 5:**
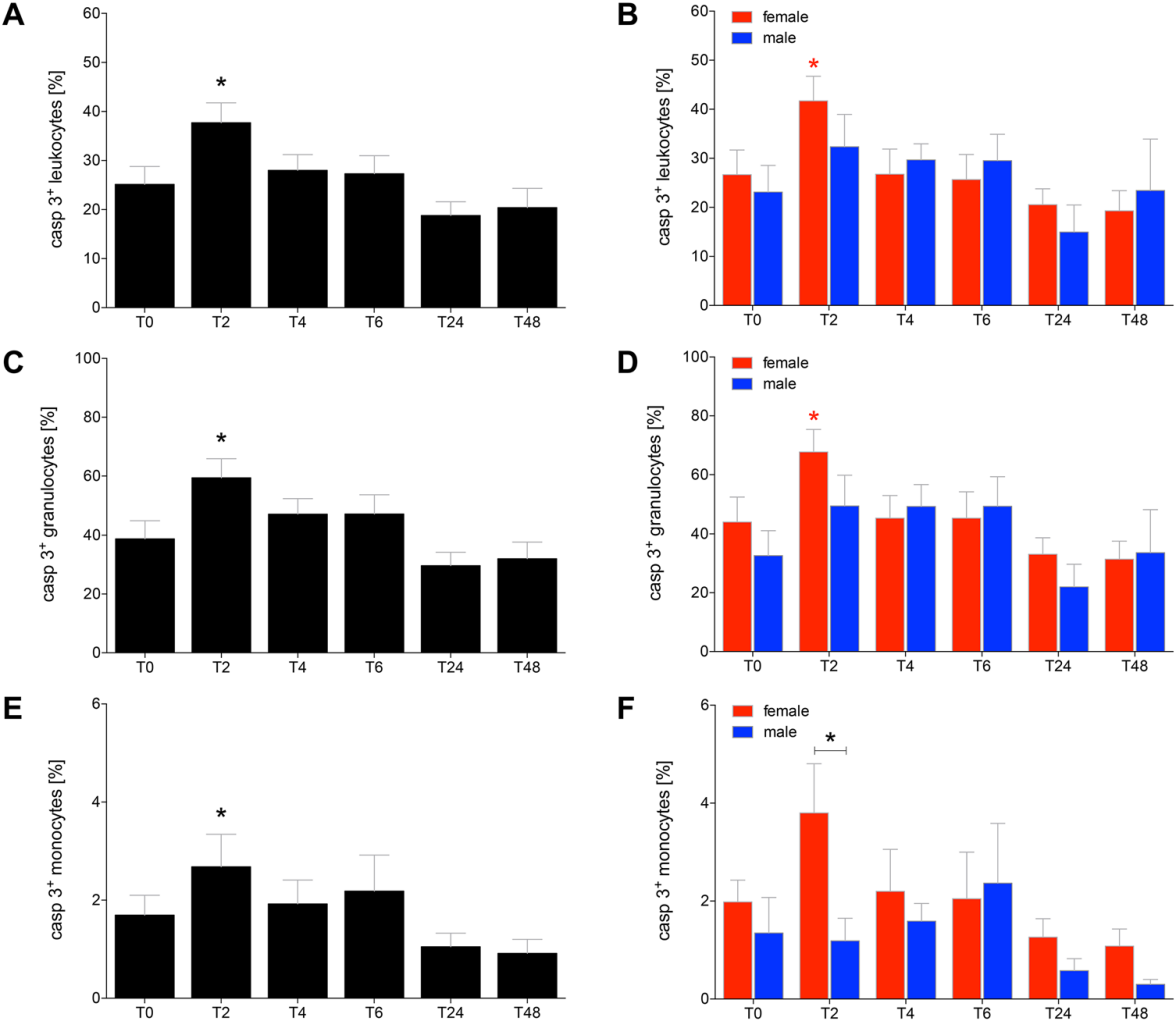
The ratio of apoptotic leukocytes (**a**, **b**), granulocytes (**c**, **d**) and monocytes (**e,** **f**) after acute alcohol drinking in healthy volunteers before, during and after drinking. The analyses of cells positive for caspase 3/7 (casp 3^+^) were performed in 12 female and 10 male healthy volunteers before *T*_0_, 2 h (*T*_2_), 4 h (*T*_4_), 6 h (*T*_6_), 24 h (*T*_24_) and 48 h (*T*_48_) after start of alcohol consumption. The data are presented as the mean ± standard error of the mean. *p < 0.05 vs. *T*_0_ or *p *< 0.05 vs indicated groups

The proportion of caspase-3/7 positive leukocytes significantly increased at *T*_2_ in all HV and in female HV compared to *T*_0_, respectively (Fig. 5a, b, *p* < 0.05). At the following time points, the proportion of caspase-3/7 positive leukocytes decreased to a non-significant level compared to *T*_0_. The differences between the female and male HV had no significance after *T*_2_ (Fig. 5b, *p* < 0.05).

The observed apoptosis of granulocytes behaved comparable to the apoptosis proportions as observed in total leukocytes. The proportion of caspase-3/7 positive granulocytes increased at *T*_2_ significantly in all HV and in female HV at *T*_2_ compared to *T*_0_, respectively (Fig. 5c, d, *p* < 0.05). At the following time points, the proportion of caspase-3/7 positive granulocytes decreased also to a non-significant level compared to the starting value at *T*_0_. The differences between the female and male HV was no further significantly changed (Fig. 5d, *p* < 0.05).

A similar distribution among the proportion of the caspase-3/7 positive monocytes as observed in total leukocytes and granulocytes at *T*_2_ was observed (Fig. 5e, f).

**Fig. 6:**
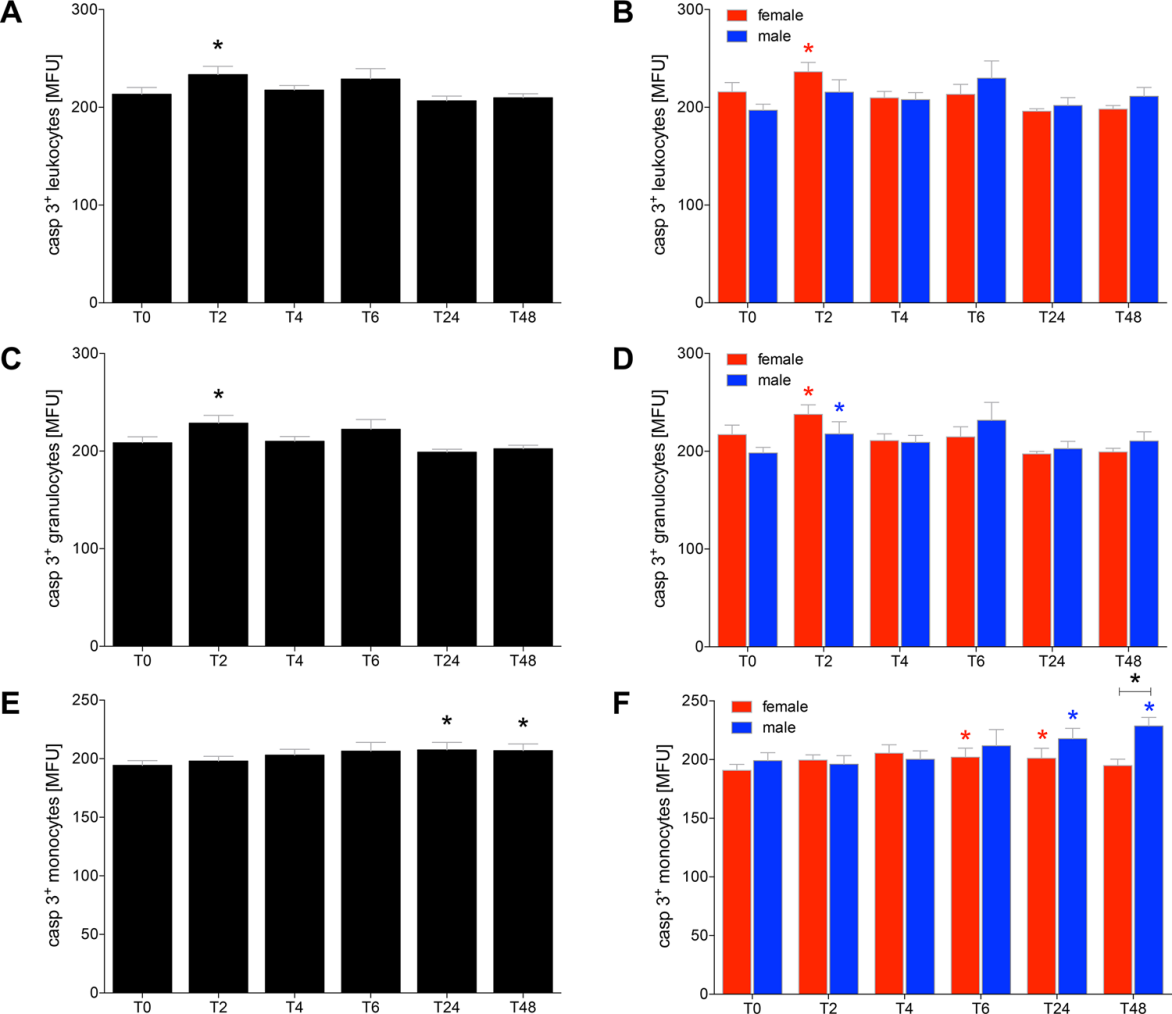
The signal induction of apoptosis by quantitative determination of active caspase-3/7 in leukocytes (**a**, **b**), granulocytes (**c**, **d**) and monocytes (**e**, **f**) after acute alcohol drinking in healthy volunteers before, during and after drinking. The analyses of mean fluorescence units (MFU) as the capacity measure for apotosis were performed in 12 female and 10 male healthy volunteers before *T*_0_, 2 h (*T*_2_), 4 h (*T*_4_), 6 h (*T*_6_), 24 h (*T*_24_) and 48 h (*T*_48_) after the start of alcohol consumption. The data are presented as the mean ± standard error of the mean. *p < 0.05 vs. *T*_0_ or *p *< 0.05 vs indicated groups

The fluorescence intensity of caspase-3 significantly increased at *T*_2_ in total leukocytes of HV and in female HV at *T*_2_ compared to *T*_0_, respectively (*p* < 0.05, Fig. 6a, b). In the following observational period there were no further significant differences in the fluorescence intensity of caspase-3.

The fluorescence intensity in monocytes significantly increased at *T*_2_ in HV as well as in both female and male at *T*_2_ compared to *T*_0_ (*p* < 0.05, Fig. 6c, d, *p* < 0.05).

With regard to caspase-3/7 positive monocytes, at *T*_24_ and *T*_48_ the increase in the intensity of caspase-3/7 expression was significant in total HV compared to *T*_0_ (*p* < 0.05, Fig. 6e). In female HV, the expression intensity of active caspase-3/7 in monocytes was significantly increased at *T*_6_ and *T*_24_, while it male HV it was enhanced at *T*_24_ and *T*_48_ compared to *T*_0_, respectively (*p* < 0.05, Fig. 6f). At *T*_48_ the intensity significantly increased in male HV compared to female at *T*_48_ (*p* < 0.05, Fig. 6f).

## Discussion

Previous studies have suggested that acute alcohol consumption may lead to the development of immunological tolerances against DAMPs and PAMPs, as shown e.g. by a reduced reaction of the immune system to antigens such as lipopolysaccharide [14, 15]. To further elaborate the impact of acute alcohol intoxication on the immunological background, we investigated the cellular functions including ROS production, phagocytosis and apoptosis of circulating leukocytes upon acute alcohol consumption in a time-dependent manner in healthy volunteers.

Our study has shown a significant increase in total leukocyte counts immediately in the first 4 h after drinking, while the cell counts recovered after 6 h to the baseline level. This initial stimulating effect of alcohol was also shown by Afshar et al. [10], that also has demonstrated the impact of binge drinking on the immune system of HV. Thus, the initial increase in leukocyte counts at two and four hours after drinking was paralleled by an early decrease in monocyte and granulocyte rates six hours later. In clinical trials, it was also shown that non-trauma patients with acute alcohol intoxication had a lower count of granulocytes [23]. This suggests that alcohol consumption affects the cell counts of both HV as well as patients. Further experiments are necessary to determine the differences and commons between those groups. However, the ratio of granulocytes and monocytes is apparently affected by alcohol, acute alcohol intoxication may trigger the production as well as the recruitment of leukocytes. These findings underline the reports of Afshar et al. showing initial inflammation upon alcohol binge drinking that was followed by anti-inflammatory effects [10]. Previous studies done by Parlesak et al. demonstrated a suppressive effect on the ROS-production in human granulocytes and monocytes which were isolated from the blood of HV [15]. On the other hand, enhancing effects of an acute alcohol intoxication on ROS production were demonstrated by Bailey et al., in rodent hepatocytes and Stadelbauer et al. in cells obtained from HV [24, 25]. However, chronic exposure to alcohol enhanced ROS production in monocytes [26, 27]. We could also confirm that acute alcohol intoxication reduced the ROS production of leukocytes, including both monocytes and granulocytes. Interestingly, the ROS-reducing effects of alcohol were observed also after 24 h in monocytes, demonstrating prolonged suppressive influence of an acute intoxication with alcohol. The alterations regarding the ROS production during the observational timeline may be in line with findings reported by others demonstrating that the ROS production in human Chang liver cells depends on concentrations of alcohol to which the cells were exposed [28].

With regard to the phagocytizing capacity of leukocytes, acute intoxication of HV with alcohol had also diminishing effects on the phagocytic activity of leukocytes in the early phase of drinking. Interestingly, the percentage of phagocytizing leukocytes decreased at *T*_4_ and *T*_6_ without having any significant impact on the capacity of active cells to phagocytize. This may be explained by a potential compensated response by the remaining cells since the leukocyte counts increase immediately during drinking. A similar study design which also included HV showed also that acute alcohol drinking had only a minimal effect on phagocytizing activity of monocytes and granulocytes [25]. Another study, investigated specifically granulocytes which were isolated from HV demonstrated a decrease in the phagocyting activity that is in line with the diminished amount of phagocyting granulocytes in our study [29]. Moreover, a suppressive effect of high alcohol concentrations in the digestion of DNA fragments in monocytes which were isolated from HV is described [30]. Thus, taken together, these results are not contradictory to our findings, as we were also able to demonstrate an early decreasing effect of an acute alcohol intoxication on the phagocyting behavior of leukocytes, albeit only to a reduced extent. Interestingly, the difference between female and male HV in terms of the phagocyting capacity was even present before drinking alcohol. Notably female HV had a higher phagocyting activity that male. Aldebert et al. have investigated the gender-specific differences in phagocyting cells from HV. According to their study, there were no differences in the count of phagocyting monocytes between female and male individuals [31]. However, the authors did not investigate the intensity in their study, and although we have observed some differences among the percentages of phagocyting cells upon stimulation between female and male, the capacity of cells to phagocytosis has shown the most prominent effects. Thus, acute alcohol intoxication is associated with reduced capability of leukocytes to phagocytosis as well as to the production of ROS.

Interestingly, the % of apoptotic leukocytes including granulocytes as well monocytes enhanced immediately upon drinking, and this effect was caused by enhanced apoptosis in cells obtained from female HV. Such pro-apoptotic effects of inflammatory immune cells upon an acute exposure to alcohol were already shown before [32, 33] and underline the theory that alcohol exerts anti-inflammatory effects on immune cells [10]. However, here we could show that the pro-apoptotic effect of alcohol is present immediately upon drinking. Furthermore, although the number of apoptotic monocytes apparently does not markedly change further after 24 or 48 h upon drinking, the activity of caspase-3/7 was enhanced in those cells that were positive for apoptosis. Already at the beginning of consumption, alcohol induced increased apoptosis in leukocytes, and thus weakened the immune system. This result may also be associated with later observed diminished proportions of granulocytes and monocytes as well as their reduced cellular functions, and provide explanation for the anti-inflammatory effects of acute alcohol intoxication. Although it was reported before that ROS also play an important role in programmed cell death [34, 35], the ROS production was rather reduced while apoptosis was induced. This discordance in observations may be due to the pro-apoptotic effects of alcohol [32] which may initially predominate.

Taken together, acute alcohol intoxication decreases the cellular functions of granulocytes and monocytes, which represent the most important players of the innate immune system in the defense against DAMPs, PAMPs and infections. This decrease may be caused by enhanced apoptosis rates of immune cells, potentially affecting subsequently also their functions with less efficient ROS production and phagocytizing activity. The weakening of phagocytosis and ROS-production can have a significant clinical impact. However, it could be shown before that a reduced phagocyting activity in polymorphonuclear leukocytes correlated with a worse outcome of septic patients [36]. Thus, an alcohol-induced reduction of phagocytosis and ROS production may lead to an enhanced susceptibility to infections, that has already been demonstrated in several studies [29, 37]. It remains considerable that binge drinking influences the immune competence of the innate immune system immediately during drinking, and furthermore, that this influence of alcohol even exists up to 2 days after drinking. Interestingly, the significant differences in the immune response between female and male remain to be further elucidated in future studies.

### Strengths

The extraordinary strength of this study is that it presents a very realistic model of binge drinking in HV. The subjects started at the evening with drinking and the consumption occurred over an extended period of time and BAC increased steadily. This setting is very common and represents a realistic way of getting drunk. It was also useful to determine alcohol-caused changes in the function of the observed parameters in a time- and dose-depended manner and can easily be transferred to other populations e.g. in elder person, to determine the differences in the response to alcohol between young and elder populations. Furthermore, our study also portrays the gender-based differences. This aspect has only been taken into account by a few studies so far but is nevertheless highly relevant.

### Limitations

Within our wide experimental design, we did not have the capacity to study the functionality of the immune defense against vital microorganisms. For example, our phagocytosis-assay shows only how many cells are functionally phagocyting but it cannot give a clue about the quality of the phagocytosis. It does not allow any statement about the digestion by the phagocyting cells. To complete the state of knowledge here follow-up studies which investigate the ability of leukocytes to kill vital bacteria by phagocytosis and ROS formation should be performed.

## Abbreviations

BAC: Blood alcohol concentration
BSA: Bovine serum albumin
DAMP: Damage-associated molecular patterns
FACS: Fluorescence activated cell sorter
HV: Healthy volunteers
IL: Interleukin
ISS: Injury severity score
MFU: Mean fluorescence units
PAMPs: Pathogen-associated molecular patterns
PBS: Phosphate buffered solution
ROS: Reactive oxygen species
*T*: Time point (hours)
